# Multifaceted, Cross-Generational Costs of Hybridization in Sibling *Drosophila* species

**DOI:** 10.1371/journal.pone.0080331

**Published:** 2013-11-12

**Authors:** Erin M. Myers, Tiffany I. Harwell, Elizabeth L. Yale, Abigail M. Lamb, W. Anthony Frankino

**Affiliations:** Department of Biology and Biochemistry, University of Houston, Houston, Texas, United States of America; University of Massachusetts, United States of America

## Abstract

Maladaptive hybridization, as determined by the pattern and intensity of selection against hybrid individuals, is an important factor contributing to the evolution of prezygotic reproductive isolation. To identify the consequences of hybridization between *Drosophila pseudoobscura* and *D. persimilis*, we estimated multiple fitness components for F1 hybrids and backcross progeny and used these to compare the relative fitness of parental species and their hybrids across two generations. We document many sources of intrinsic (developmental) and extrinsic (ecological) selection that dramatically increase the fitness costs of hybridization beyond the well-documented F1 male sterility in this model system. Our results indicate that the cost of hybridization accrues over multiple generations and reinforcement in this system is driven by selection against hybridization above and beyond the cost of hybrid male sterility; we estimate a fitness loss of >95% relative to the parental species across two generations of hybridization. Our findings demonstrate the importance of estimating hybridization costs using multiple fitness measures from multiple generations in an ecologically relevant context; so doing can reveal intense postzygotic selection against hybridization and thus, an enhanced role for reinforcement in the evolution of populations and diversification of species.

## Introduction

Predicting the evolutionary fate of incipient, new or even established species challenged with sympatry requires knowledge of the fitness consequences of hybridization. Complexes of potentially hybridizing species offer the opportunity to identify the ecological processes and proximate mechanisms that underlie natural and sexual selection acting on hybrids and backcross progeny. Consequently, such species complexes are well-suited for studies of species formation, the maintenance of species boundaries and in particular, of reinforcement - the process by which natural selection against hybrids strengthens prezygotic reproductive isolation [1–5]. Theoretical and empirical work regarding the process of reinforcement is predicated on the strength of selection against hybrids [3,6,7]; selection against hybrids can lead to enhanced species discrimination in geographic regions where hybridization is likely, accelerating differentiation between species [3,8] (e.g. [9–11]). Indeed, this prediction has been borne out in experimental populations subject to complete selection against hybrids [12–16]. However, hybrids often have intermediate or variable fitness, producing a more variable evolutionary response to selection against hybridization in natural and experimental settings [10,17–25]. 

The evolutionary outcome of interactions between potentially hybridizing species will be determined largely by the fitness of hybrids relative to the parental species [5,7,26]. Historically and frequently out of necessity, many studies quantifying hybrid fitness focus on one or two fitness components estimated from one point in the lifecycle (e.g., [27,28]). Such studies are informative; particularly when the fitness component studied has a large effect (e.g. [27]) or is strongly correlated with lifetime reproductive success [29]. However, estimates of hybrid fitness can depend on the traits examined, when during ontogeny they are assessed (e.g., [17]), and the environment in which the measures were made [21,30,31]. For instance, hybrid offspring may have low fecundity but high viability relative to progeny from intraspecific matings (e.g., [25]). In such situations, different fitness components may yield a range of fitness estimates, but potentially all are important as it is their net contribution that ultimately determines the evolutionary consequences of hybridization. Thus, researchers are beginning to examine multiple fitness components in F1 hybrids [25,29,32,33]. However, although some fitness effects are manifest in F1 hybrids such as hybrid sterility in one sex (e.g., [34]); additional fitness decrements may be expected in F2 or backcross progeny (e.g., [7,35]). Estimating the full strength of selection against hybridization therefore relies on including the contributions of many fitness components across multiple generations. Only a handful of studies have examined fitness components throughout the lifecycle (e.g., [25,33,36]) or across generations (e.g., [7]), and all found multiple sources contributing to considerable fitness costs of hybridization. To our knowledge, however, no single study has examined multiple fitness costs of hybridization across more than one generation.

Extreme fitness reductions, such as hybrid inviability or sterility in one sex, are sufficient to induce reinforcement on their own [9,37] and much of the literature has focused on these intrinsic postzygotic incompatibities [3], yet, reduction in other postzygotic fitness components may also facilitate and strengthen the process [3,18,33]. Focusing attention solely on such sources of extreme fitness effects may underestimate the total force of selection against hybrids and thus provide an incomplete picture of the selective process. Indeed, some systems may experience reinforcement without such single, large costs [3,38]. For example, partially recessive alleles in the parental species may be expressed in hybrids or backcross progeny, decreasing viability, lowering developmental robustness, causing sterility or otherwise lowering fitness [18,39,40]. Reductions in fitness can also occur when co-adapted gene complexes within species are dissociated or loci incompatible between species are brought together in hybrids or backcross progeny [7]; indeed, such incompatibility loci can be used advantageously to generate mapping populations to identify the genes responsible for extreme fitness reducing traits such as hybrid sterility (e.g., [40–42]). Finally, fitness costs can result from the generation of hybrid progeny with intermediate phenotypes that suffer increased natural (e.g. [31,43]) or sexual (e.g. [44]) selection. Thus, hybridization can cause fitness decrements in many ways, over time strengthening barriers to gene flow between species [7,18,45,46]. Consequently, it is important to consider the full range of fitness effects in both F1 hybrids and backcross progeny. Inclusion of such fitness costs of hybridization may increase the range of conditions under which reinforcement can be seen to play an important evolutionary role and reduce reliance on hybrid inviability or sterility as the sole selective force(s) in this process [25,26].

Utilizing *Drosophila pseudoobscura* and *D. persimilis*, a sibling species pair used as a model for speciation and reinforcement studies for over 75 years [1,9,12,47,48], we quantify the fitness decrement imposed on hybrids by a variety of intrinsic and extrinsic factors in both the first generation hybrids and their backcross progeny. We assess several fitness components: offspring viability, larval competitive ability, lifetime fecundity, frequency of developmental abnormalities in adults, and adult flight performance and combine these measures to estimate the total fitness consequences of hybridization across generations relative to purebred progeny from each species. In general, consistent with the ongoing reinforcement in this species pair [9], we predicted that hybrid and backcross progeny would have lower relative fitness than parentals across the suite of measures. We also predicted that this effect would be most pronounced in males in accordance with Haldane’s rule [49–54]. Our results describe the complex fitness costs associated with hybridization in this species pair and indicate that post-zygotic selection against hybridization is much stronger than that imposed solely by hybrid male sterility - nearly twice as strong, acting on both males and females in both F1 and backcross generations. Such strong selection against hybrids likely enhances the conditions under which reinforcement shapes species boundaries.

## Methods

### Study System

**Figure 1:**
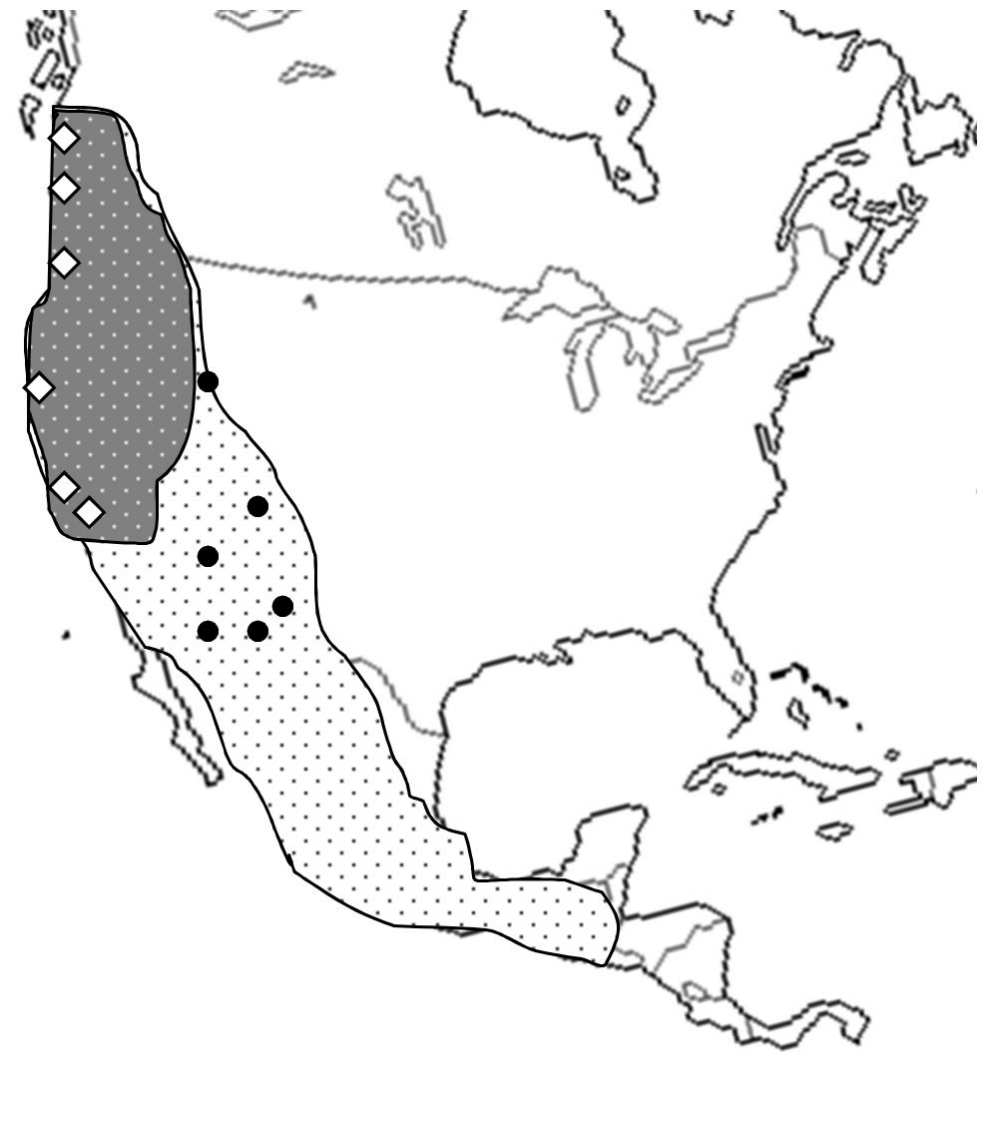
*D. pseudoobscura* (white) and *D. persimilis* (gray) ranges and populations sampled to create the synthetic outcrossed parental species (PS) populations. Geographically proximate populations are shown as a single dot. See Table S1 in File S1 for lineage and locality details.

The sibling species pair *D. pseudoobscura* and *D. persimilis* is a well- studied biological model for studying speciation, reinforcement and species persistence [1,9,12,34,47,55–63]. These partially sympatric species, with the range of *D. persimilis* nested within part of the range of *D. pseudoobscura* (Figure 1), diverged ~850,000 to 500,000 years ago [64,65]. Hybridization in nature occurs at low frequency and backcrossing by F1 hybrid females is rare [55,66]. Geographic patterns of mate preference within *D. pseudoobscura* indicate that reinforcement is ongoing [9]. Species discrimination is weaker in females from populations allopatric to *D. persimilis* than in females from populations sympatric to *D. persimilis*, and the degree of discrimination varies among these sympatric populations [60]. Hybridization between *D. pseudoobscura* and *D. persimilis* produces infertile males but reproductively capable hybrid females [9,34,41,67]. Studies of reinforcement in this pair have focused on hybrid male sterility as the selective agent (e.g. [9,18,37]). Although additional work has studied further aspects of hybridization fitness (e.g., [17,35]), a wider assortment of selective forces acting against hybridization in this system has not been systemically characterized within and across generations, and it seems probable that additional, important reductions in F1 hybrid or backcross progeny fitness are likely. 

### Creating the Experimental Parental Populations

We generated synthetic populations of *D. pseudoobscura* and *D. persimilis* by combining geographically and genetically distinct lineages taken from throughout the species range for *D. persimilis* and from throughout the allopatric range of *D. pseudoobscura* in the United States [68,69] (Figure 1, Table S1 in File S1). Lineages were acquired from the Drosophila Species Stock Center, except for one (MSH) donated by M. Noor (Table S1 in File S1). By combining lineages from across the species' ranges, we hoped to increase the pool of available genetic and phenotypic diversity and consequently reduce any fitness decrements resulting from inbreeding during stock culture (similar to [12,37]). To create the populations, all lineages for each species (N=10/species) were crossed in a full-factorial design over four generations until the offspring were a genetic mosaic of all starting lineages. Factorial crosses were conducted between 20 males and 20 females in triplicate in each generation. Because *D. persimilis* and *D. pseudoobscura* are not readily distinguishable from each other or their hybrid progeny, unique recessive eye-color mutations were introduced into each synthetic population at generation five [12]. *D. pseudoobscura* received a *vermillion* mutation (*v*), giving it a bright red eye color whereas *D. persimilis* received a *sepia* mutation (*se*), producing dark purple eye color and yellow testes. Both mutations are X-linked in these species and therefore, as the mutations are recessive, hybrid females have wild-type eyes. We conducted behavioral screens of these mutants to ensure that courtship and mating behavior is not significantly different than wild-type flies. Briefly, a single unmated female was placed in a vial with an unmated male and the cotton plug pushed down to leave approximately one inch of space. Flies were observed from the onset of the experiment until courtship began (if within five minutes) and for five minutes following the onset of courtship to determine if copulation was attempted. Data were recorded on courtship latency, copulation attempts and no significant differences were observed using ANOVA between males with or without the eye color mutations (*D. persimilis* males: courtship latency F_1,67_=1.8036 p=0.1838, copulation attempts F_1,67_=0.1979, p=0.6579; *D. pseudoobscura* males: courtship latency F_1,77_=1.8002, p=0.1836, copulation attempts F_1,77_=2.2451, p=0.1381; for complete methods see 63). The fitness effects of these mutations in these or other related species is not appreciable (*sepia* [70–73]:, *vermilion* [71,74]:). The synthetic populations were maintained as large (N > 1000 flies) free mating colonies in 32.5cm cubes (0.034m^3^) for at least five generations before sampling flies for the experiments described below. Unmated adults were collected from these populations to generate three population classes (parental species, PS; F1 hybrid, F1; and first generation backcross progeny, BC) for each experiment below. For each population class, we assessed several fitness components: larval survival and competitive ability, fecundity, frequency of developmental abnormalities, and adult flight performance. We combined these measures to estimate the total fitness consequences of hybridization from the initial hybridization event and one generation of backcrossing. 

### Rearing Conditions

All flies were reared on a sucrose-dextrose-yeast-agar diet [63]. They were maintained on a 12:12 light cycle at 20°C with 75% humidity. Flies from the experimental parental populations were maintained in 32.5cm cubes with several bottles with 25mL of fly food. F1 and BC progeny were reared in standard vials with 7mL food at moderate larval density (~50 eggs/vial), resulting in low variation in adult body size. This low density is standard in our laboratory as it avoids competition and produces high survivorship to adulthood [63,75].

### General Experimental and Statistical Approach

**Figure 2:**
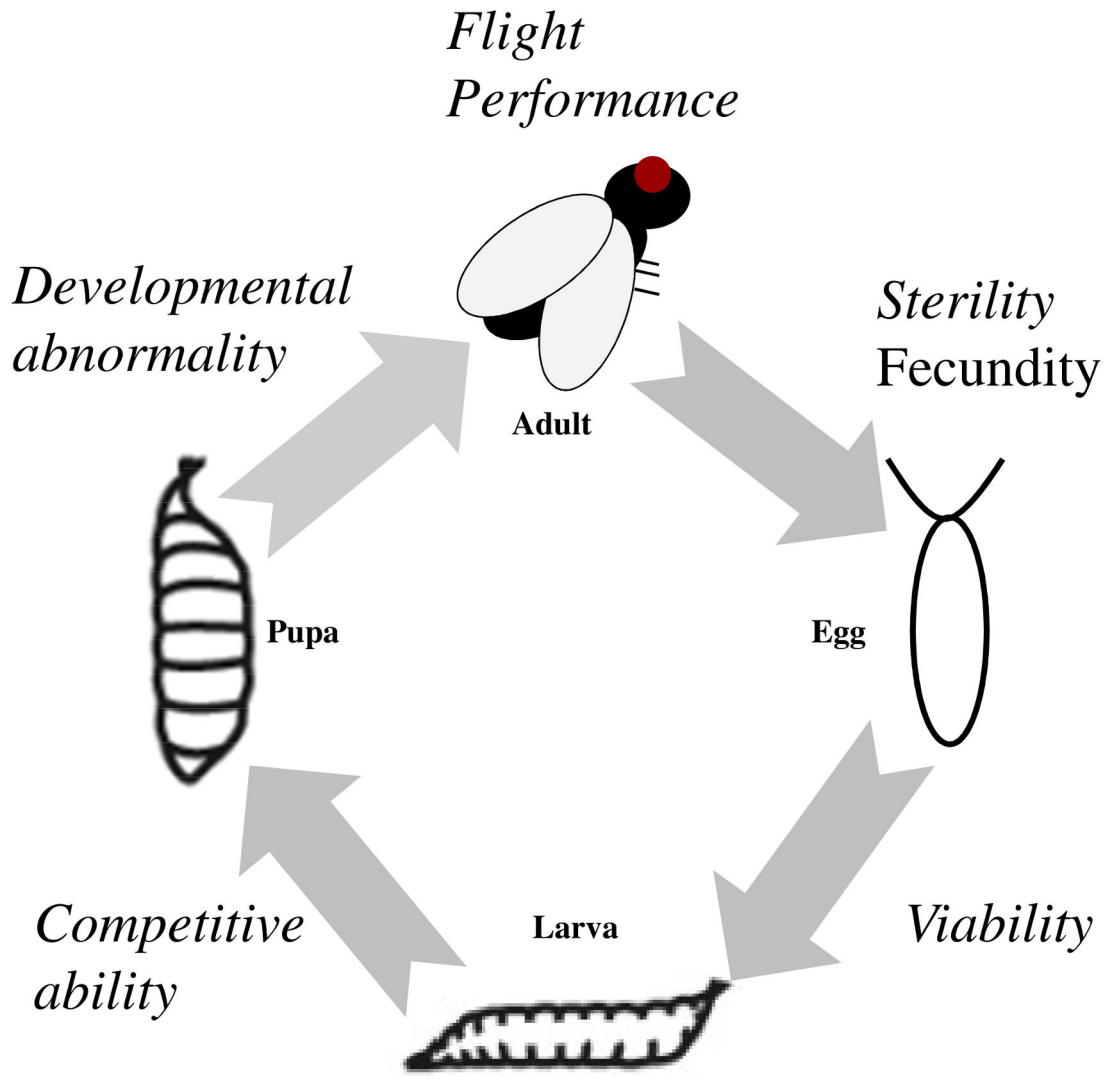
Lifecycle of *Drosophila*. Fitness of F1 hybrid and backcross progeny relative to the parental species was assessed for several traits throughout the lifecycle of the fly. Traits for which a significant fitness effect was seen are shown in italics.

Our goal is to use several fitness components measured in two generations to produce composite fitness estimates for hybrids, backcross and pure-bred flies. Our fitness components (see below) were selected to represent a diverse suite of traits that are important for fitness across the lifecycle of the fly (Figure 2), some of which have been assessed in other studies (e.g., [17,33,35,84]) whereas others are novel. The fitness components we assay can be broadly divided into categories, those resulting in relatively direct effects on fitness (e.g. fecundity and competitive ability) versus those that have a relatively indirect effect on fitness (e.g., flight performance and developmental abnormalities). 

In the experiments that follow, the crossing schemes produce progeny from all possible crossing directions (e.g., hybrid progeny in the experiment are the product of reciprocal *D. persimilis* and *D. pseudoobscura* crosses), as cross direction may affect progeny fitness (e.g., [33,41]). Progeny from such crosses comprise subpopulations within a class (e.g., subpopulations within the class of hybrid progeny or group of backcross progeny), and exist in these designs as their own experimental units. However, as we are concerned primarily with the overall cost of hybridization rather than teasing apart which specific paths to hybridization are the most costly, we designed our experiments such that our results would be based on equal sampling of each cross-type for each fitness measure. Thus, we allocated our sampling effort in a manner emphasizing subclass representation to build a robust fitness component estimate for each class at the necessary expense of investing effort in estimating components within each cross-type. Thus, by design, data from subclass replicates and cross directions are pooled within classes for analyses. Moreover, pooling is justified as there were generally few significant differences among cross-types (Table S2 in File S1). Similarly, we present the pooled results for the parental species as there were no significant differences in fitness between them for most measures (Table S2 in File S1). In this manner, we ensure that our findings result from equally weighted contributions of all possible cross-types, meaning our tests for fitness differences among classes are conservative and differences identified between hybrid, backcross or parental species are robust. In general, we predict that the parental species will have higher fitness relative to either the hybrid or backcross progeny, consistent with reinforcement.

### Fitness Component Assay 1 (Direct Fitness) – Egg to Adult Viability

Offspring viability is commonly used to assess selection against hybridization (e.g. [17,18,33,76]). To assess the viability of offspring from egg to adult in the absence of competition, we collected eggs from each population type (PS, F1, BC) from all cross directions. We ensured that all possible hybrid types were included by utilizing eggs from each cross direction for the F1 and BC crosses (two crosses for F1 and four crosses for BC; Table S2 in File S1). We placed 50 eggs from each cross type and direction in vials containing 7mL fly food. This low density is standard in our laboratory as it avoids competition and produces high survivorship to adulthood [63,75]. There were three replicates for each parental species and each direction of the F1 and BC crosses Eggs were incubated at 20°C and the number of eclosing adults compared among classes. Because eggs were collected in two blocks (PS/F1 and PS/BC), blocks were analyzed separately to test for differences between population classes. We used a one-way, fixed effect ANOVA to test for differences between PS and F1classes in the number of eclosed flies. For PS and BC classes we used a Wilcoxon signed rank test to test for differences in the number of eclosed flies [77]. We tested for differences in the sex-ratio among classes in a similar fashion using the Wilcoxon test. 

### Fitness Component Assay 2 (Direct Fitness) - Larval Competition

Even if F1 or BC offspring are viable, the larvae may suffer a reduction in fitness if they are competitively inferior to their single-species counterparts [17]. Thus, we formulated two experiments to test the ability of larvae to compete for limited nutrient resources. For both competition experiments, viability to adult eclosion was determined at three levels of competition, Low (50 eggs/vial), Medium (100 eggs/vial), and High (200 eggs/vial), with 50% of eggs in a vial drawn from each competing class, consistent with density ranges in other Drosophilid experiments (e.g. [78]). Each competitive paring was replicated nine times at each competition level (Class totals for each competition level, PS: N=18, F1: N=18, BC: N=36). Using an ANOVA model, we tested for effects of density, population class, and their interaction on the proportion of flies surviving from egg to adulthood. To keep the models simple, we analyzed each block and experiment separately. 

In the first experiment, we placed PS, F1 or BC larvae into competition against the larvae of *D. pseudoobscura* OR-PX (hereafter OR-PX) and quantified the number of flies that eclosed. OR-PX carries both an eye color (*orange*) and wing vein mutation (*plexus*) allowing it to be distinguished easily from the PS, F1 or BC progeny by the presence of additional cross-veins [79]. Because OR-PX is a weak competitor (Myers, unpublished data), this experiment established baseline competitive ability of each offspring class while avoiding interactions among focal classes. This experiment was conducted in two blocks such that PS and F1 larvae were used in one block, and PS and BC in the second.

In the second experiment, we competed F1 larvae from one cross direction directly against one of the PS species, (F1 hybrid [*D. persimilis* males x *D. pseudoobscura* females] competed against *D. persimilis* or F1 hybrid [*D. pseudoobscura* males x *D. persimilis* females] competed against *D. pseudoobscura*) and quantified the number of eclosing flies from each class. Competitive pairings were constructed in this manner to ensure the identification of F1 and PS males using our phenotypic markers. 

### Fitness Component Assay 3 (Direct Fitness) - Fecundity

Hybridization affects fecundity differently among systems (e.g., [17,80,81]) and thus may impact the action of reinforcement. We quantified the effects of hybridization on fecundity by assessing the average egg production of PS, F1, and BC females over three months. For PS, 10 females of each parental species were paired conspecifically with 10 males of the same species, while 10 F1 or BC females were paired with 10 males (5 from each of the two species) for all possible F1 and BC cross directions. Each possible set of 20 flies was replicated three times (N= 6, 6, 12 for PS, F1, and BC respectively). Flies were transferred to juice plates dotted with yeast paste three times per week. Eggs were counted once each week 24hrs after transfer to a fresh juice plate. Collection continued for 12 weeks. Flies were also surveyed weekly to track female mortality and generate estimates of per capita egg production. Additional males were added part way through the experiment to ensure females were not sperm limited (e.g. [17]). Fecundity was estimated as the total egg production of a given replicate. A one-way ANOVA was used to test for differences in the reproductive output of PS, F1, and BC females. 

### Fitness Component Assay 4 (Indirect Fitness) - Frequency of Developmental Abnormalities

**Figure 3:**
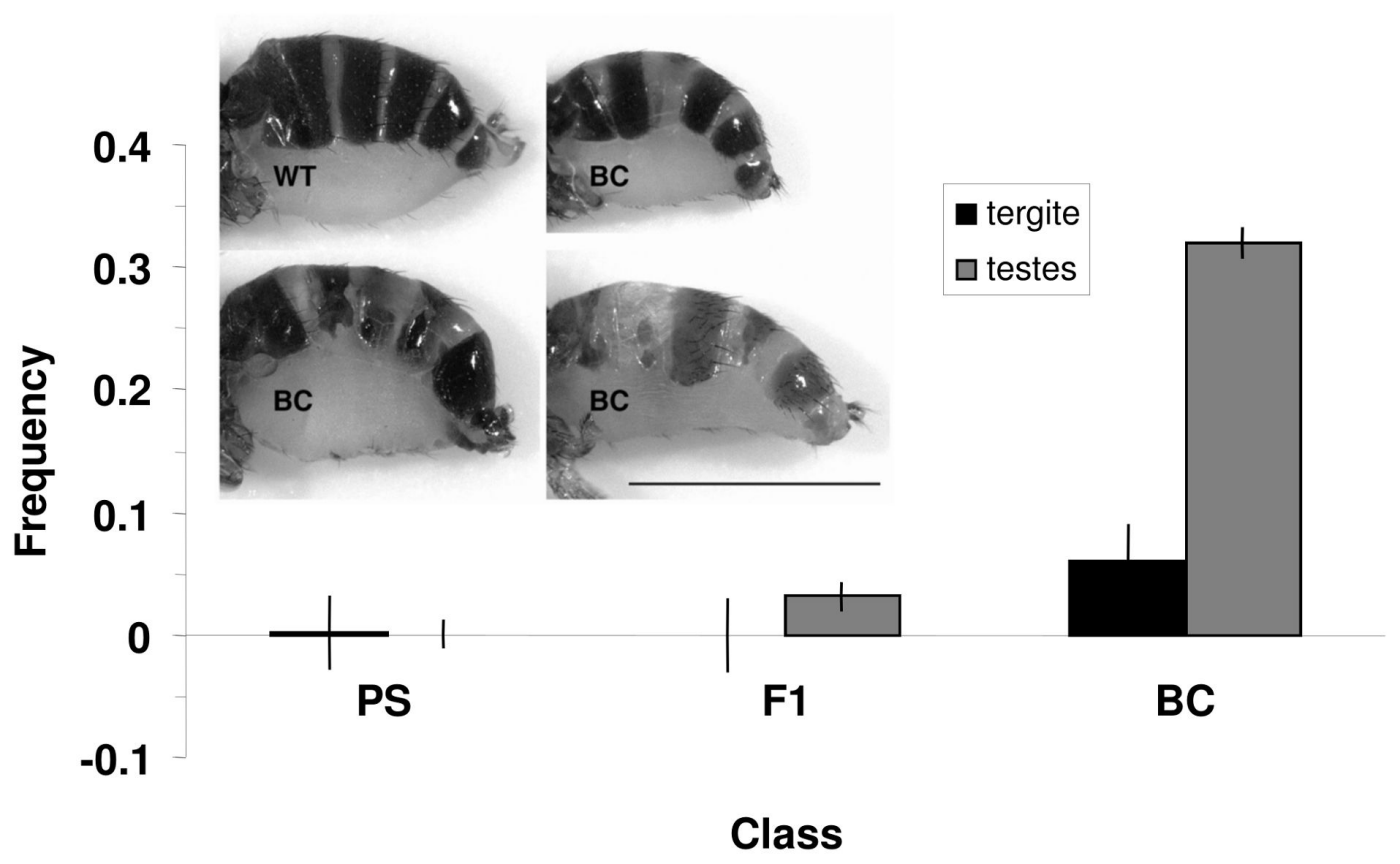
Frequency of developmental abnormalities by offspring class. Abnormal abdominal tergites (black) and reduced testes size (gray) are shown as a function of their frequency in Assay 4 (+/- 1 SE). Inset shows examples of wildtype (WT) and abnormal tergite expression (BC). Scale bar 1mm.

Male F1 and backcross hybrids in these and other *Drosophila* *spp.* are frequently characterized by exceptionally small, non-functional testes [41,82] and an increased frequency of visible abnormalities in backcross progeny has been suggested in this species pair [35]. Such abnormalities could produce a cost to hybridization directly, and are likely indicative of generally reduced developmental stability which may further reduce fitness indirectly. We quantified the frequency of flies with visible abnormal abdominal tergites and reduced testes in PS, F1, and BC progeny. Each cross type (see General Approach above) was replicated three times and 50 eggs were collected from each replicate for three days for a total of N=9 food vials for each cross type and reared at 20°C. Eclosing adults were examined for abnormal tergites (Figure 3) and reduced testes by the same observer (EMM) to minimize observer variation in interpretation of testes size.

For the composite estimate of fitness (see below), we compared the frequency (rather than the absolute number) of flies with developmental abnormalities among groups to account for variability in the number of flies that eclose both and among classes and between replicates within classes. The frequency of reduced testes size was calculated using only the number of males that eclosed from a vial whereas abnormal tergite frequency was calculated using the total number of flies that eclosed. To analyze differences in the frequency of these abnormalities among population classes we used separate nominal logistic regression analyses. For reduced testes, we tested for effects of population class on the probability of having a mutation, while for tergite abnormalities we used a model that also included sex and a class by sex interaction. Finally, we compared odds ratios to test for significant differences among groups.

### Fitness Component Assay 5 (Indirect Fitness) - Flight Performance

Flight performance can affect fitness by influencing an individual's ability to disperse, find a mate, locate food, avoid predation, etc., [83]. We quantify upwind flight performance using a custom, progressive wind tunnel similar to that used to phenotype flight ability by Weber [84,85]. Briefly, the tunnel measures the maximum upwind flight ability of *Drosophila* populations. The tunnel consists of a series of smooth, 12cm passageways that connect 15 collection chambers, into which the flies self-sort based on their ability to overcome the headwind. At each collection chamber, the passageway headwind steps up 2 cm/sec, producing a range of 0-30 cm/sec headwind over the span of the tunnel. The passageways create a prolonged flight challenge at each headwind speed; airborne flies progress through the passageways until reaching their maximum upwind flight ability, at which point they are blown into the nearest collecting chamber, where they remain until the trial ends. Flies are motivated to move through the tunnel, against the increasing headwind, via a combination of positive chemotaxis towards a liquid yeast scent gradient originating at the strong headwind end of the tunnel, positive phototaxis towards an upwind light source, and negative geotaxis (the strong headwind end of the tunnel is elevated 35° above the horizon). To conduct a flight assay, up to flies are released *en mass* at the low-headwind end (0cm/sec) of the tunnel; the flies self-sort based on fight ability for 30 min, at which time the tunnel is flooded with CO_2_ and the flies collected from the chambers. We can mark groups of flies by feeding them food dyed with unique colors, enabling the simultaneous, rapid testing of large, mixed populations of flies. The distribution of individuals in the collection chambers reveals the flight ability of the population. Repeatability of flight performance (maximum flight capacity of the first and third quartile of 20 different *Drosophila* *spp.* lineages across three replicate trials) is high for a behavior (*r* ~0.6; W. A. Frankino, unpublished data). 

In two replicate runs, we flew males and females *en masse* from the PS (N=88 males, 229 females combined total), F1 (pooled from both directions of hybrid crosses; N=121 males, 280 females combined total), and BC (pooled from all four potential crosses; N=175 males, 223 females combined total) classes. As abnormal abdominal tergites were more prevalent in the BC class (see below), we excluded individuals with these visible mutations from the test populations. *D. melanogaster* controls from a large, outcrossed population (N=72 males, 126 females combined total) were used. Males and females were flown together as previous research showed that flies generally do not interact inside the tunnel [84]. Prior to the trials, flies were fed a population-specific colored yeast paste. This colored paste is retained for several hours in the gut and thus enables identification of source population of the flies after each trial. Flights were conducted at 22°C and lasted 60 minutes. Following each run, flies were anesthetized in the chamber reflecting their maximum upwind flight performance, removed to an empty vial and frozen for subsequent identification. 

To analyze patterns in flight performance among classes, we ran separate Kolmogorov-Smirnov 2-sample tests [86] on each sex. We pooled the samples from replicates because unexpected mortality on the day of the experiment lead to uneven sample sizes between runs in some of the classes, essentially making the experiment unreplicated. Because this is a 2-sample test, we needed 3 comparisons to assess pairwise differences across classes and thus we adjusted the alpha level for significance testing using a sequential Bonferroni correction [87,88].

### Composite Estimate of Relative Fitness

To estimate the single and multi-generation cost of hybridization based on the fitness components described above, we combined their effects across life stages and generations. We determined the fitness of F1 or BC relative to PS by generating a ratio means for each fitness component (e.g. number viable F1 offspring/number viable PS offspring). The PS value was calculated as the mean across the two parental species, while the F1 and BC values were calculated as the mean of all possible cross directions (complete dataset in Table S2 in File S1). Taking the average value for the classes is valid as we interested only in the general fitness consequences of hybridization, not in the strength of selection asymmetries acting on the individual PS or those resulting from specific cross types within the F1 or BC classes. Moreover, averaging the effects of the cross-types within F1 and BC classes is valid as preliminary analyses indicated few significant effects of cross type on any response variables (Table S2 in File S1), although the lack of significant cross type effects may result in some cases from reduced power owing to low within-cross type replication. 

**Figure 4:**
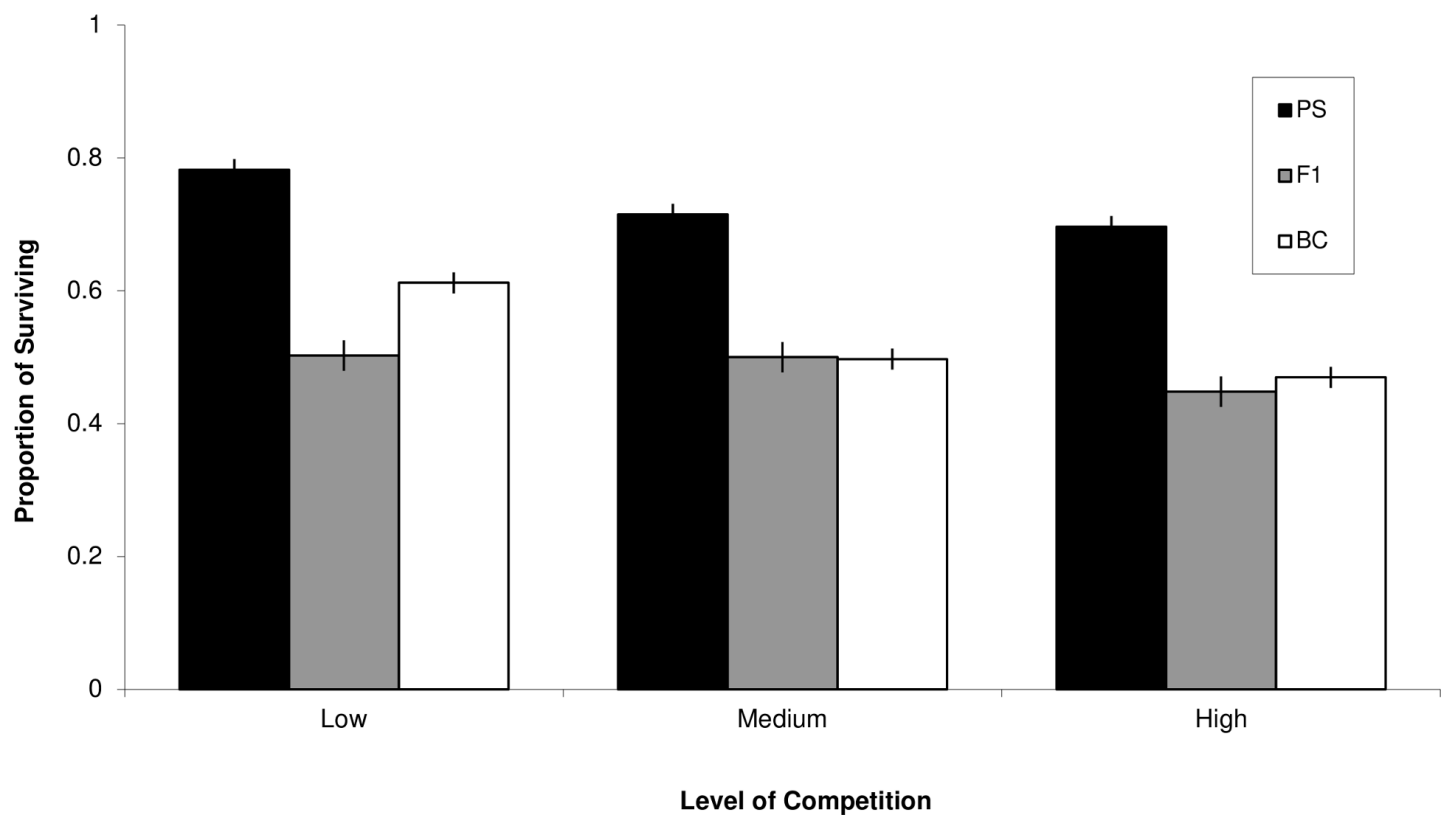
Egg to adult viability. Proportion of adult flies eclosing from parental species (PS), F1 hybrid (F1) or backcross progeny (BC) classes (+/- 1 SE) larvae in competition with larvae from the mutant OR-PX at three competition levels. The parental species are superior competitors to both hybrid and backcross larvae against the weak competitor. PS values are averaged across the F1 and BC experiments.

In the case of multiple levels of competition (Fitness Component Assay 2), the relative fitness included in the composite measure is from the highest competition treatment only. The composite estimate can also be calculated for each of the other competition levels, although variation in the composite estimate is minimal as hybrid survival was lower in all competition levels (Figure 4). Additionally, in cases of frequency of abnormality and flight performance (Fitness Component Assays 4 and 5), the relative fitness is determined by comparing the frequencies of flies without visible mutation and the frequencies of flies reaching the end of the wind tunnel (i.e., the strongest fliers). We averaged the relative fitness values for F1 and BC males for abnormal testes and abnormal tergites to use as a general estimate of abnormal development. Finally, the values used for sterility were extracted from previously published estimates of sterility [41].

The single-generation costs of hybridization were computed by multiplying the relative fitness values within each generation within classes (similar to [25]), as we are interested in the cumulative effect of hybridization. These effects, occurring over the lifecycle of a fly, are multiplicative in nature (e.g. flies experience fitness effects in fecundity and flight performance). Our fitness effects may contribute differently to lifetime fitness (e.g., the lifetime fitness effects of fecundity and flight performance likely differ), however, as the relative weights of such effects are unknown, and because we are more interested in the direction and magnitude of the relative fitness of hybrids than their precise relative fitness, we weight all estimates equally [25]. In addition to treating all fitness components equally, we divide them into direct and indirect fitness categories and then use the composite and averages of the components to assess fitness estimate conservatively which we analyze separately. 

Multi-generational effects were computed by multiplying the F1 and BC relative fitness values within groups [7]. We calculate the fitness effect first for both sexes independently, and then as a combined fitness effect within each generation; the former, single-sex estimates enabled us to examine asymmetries between the sexes in the fitness costs of hybridization. 

## Results

### Fitness Component Assay 1 – Egg to Adult Viability

F1 viability from egg to adult was not distinguishable from the PS (F_1,22_ = 0.7772, p= 0.3875; means ± s.e.; F1= 41.39 ± 1.10 and PS = 43.33 ± 1.91; N=18 and 6 vials, respectively). However, BC eggs produced fewer adults than did PS eggs (means for BC= 31.06 ± 1.43 and PS = 37.94 ± 2.00; χ^2^ = 8.773, p=.0031, N= 36 and 18 vials, respectively). Sex ratio of the eclosing flies did not differ significantly for the F1 or BC compared to PS (F1 v PS: mean PS = 1.1477 ± 0.1027 and F1= 0.978 ± 0.0593; χ^2^= 2.351, p=0.1252; BC vs PS: mean PS = 1.0707 ± 0.1145 and BC= 0.909 ± 0.0821; χ^2^= 0.9547, p=0.3285). Thus, costs of hybridization through offspring viability are not manifest in the first generation, but F1 females suffer reduced fitness through the production of less viable BC progeny.

### Fitness Component Assay 2 - Larval Competition

The weak competitor OR-PX reduced survival to adult eclosion more in F1 and BC than in the PS flies (Table 1; mean proportion surviving across competition levels ± SE: PS = 0.6959 ± 0.027, F1 = 0.4841 ± 0.027; PS = 0.7667 ± 0.019, BC = 0.5267 ± 0.013). In addition to assessing the raw counts of surviving flies, we also examined the proportion of surviving flies. We found a significant effect of population class (Table 1; Figure 4), indicating that the F1 and BC larvae are inferior to the PS against even a weak competitor. Competition level was a significant effect in the PS/BC analysis however, there was no significant interaction between competition and population class in either analysis.

**Table 1:** Analysis of variance and effect tests for population class (PS, F1, and BC) and level of competition (Low, Medium, High), as well as their interaction for the proportion of flies surviving from egg to adult in competition against ORPX.

**ANOVA for F1**					
Source	DF	Sum of Squares	Mean Square	F Ratio	p
Model	5	1.2846	0.2569	6.7474	<0.0001
Error	101	3.8459	0.0381		
Total	106	5.1306			
**Effect Tests**					
Source	N parm.	DF	Sum of Squares	F Ratio	p
Pop. Class	1	1	1.2067	31.6898	<0.0001
Level of Comp.	2	2	0.0671	0.8812	0.4175
Interaction	2	2	0.01825	0.2396	0.7874
**ANOVA for BC**					
Source	DF	Sum of Squares	Mean Square	F Ratio	p
Model	5	2.5892	0.5178	27.0106	<0.0001
Error	156	2.9908	0.1917		
Total	161	10.7264			
**Effect Tests**					
Source	N parm.	DF	Sum of Squares	F Ratio	p
Pop. Class	1	1	2.072	108.076	<0.0001
Level of Comp.	2	2	0.3957	10.3196	<0.0001
Interaction	2	2	0.015	0.3913	0.6768

Significant differences were seen for both analyses for population class, but competition level was only significant in the BC analysis.

As eye color phenotype was needed to distinguish between competitors, we analyzed cross types separately in the second competition experiment. ANOVA revealed that the outcome of competition between F1 and the PS was dependent on hybrid cross direction and the PS species against which competition occurred. Across competition levels, significantly fewer F1 than *D. persimilis* PS larvae from survived to adulthood (F_1,52_=13.4832, p=0.0006) when competed against *D. pseudoobscura*, with the effect being largest at the medium and high levels of competition. In contrast, F1 larvae in competition against *D. persimilis* did not differ significantly in the number surviving to adulthood as compared to *D. pseudoobscura* PS larvae (F_1,52_=0.0933, p=0.7612), suggesting that *D. persimilis* may be a weaker competitor than *D. pseudoobscura*. In sum, these two experiments reveal that larval competition with the either parental species reduces the fitness of both F1 and BC flies under more ecologically relevant conditions [89,90]. 

### Fitness Component Assay 3 - Fecundity

Although F1 female body size appeared qualitatively larger than PS and BC females, egg production was not significantly different among classes (means ± SE: PS = 2395.33 ± 528.60, F1 = 3286.17 ± 528.60, BC= 2922.00 ± 373.78; F_2,21_ = 0.7218, p=0.4975). Because F1 and BC females trended toward higher egg counts than PS females, contrary to our initial prediction, we examined two possible mechanisms that could contribute to the slightly higher fecundity of F1 females, female longevity and per capita egg production. There was no significant difference in female longevity among groups (F_2,21_ = 2.1534, p=0.1410), however, there was a significant difference in the per capita egg production (F_2,21_ = 3.7898, p=0.0393). F1 females had increased per capita egg production relative to the PS females (post-hoc analysis, mean difference ± SE = 16.101 ± 6.308, p=0.0468), although BC females did not differ from either PS or F1 females. Thus, we see no fecundity cost of hybridization females; however, as indicated both above and below, the quality of their offspring is reduced relative to PS offspring. 

### Fitness Component Assay 4 - Frequency of Developmental Abnormalities

The F1, BC and PS classes differed significantly in the occurrence of abnormal abdominal tergites (χ^2^=52.2126, p<0.0001; Figure 3). These differences result from the greater frequency of abnormalities in BC flies (6.1%) relative to F1 (0%; p<0.0001) and PS flies (1.6%; p<0.0001). F1 flies did not differ significantly from PS flies (p=0.9855). There were significant differences among the BC cross-types in the frequency of tergite abnormalities (χ^2^= 12.7026, p=0.0053). There was no significant difference between the sexes nor was the interaction term significant (χ^2^=4.2083e^-5^, p=0.9948; χ^2^=0.2843, p=0.8675). 

We also found significant differences in the frequency of reduced testes among males from the PS, F1 and BC classes (χ^2^=189.55, p=<0.0001; Figure 2). As with other analyses, we found no significant differences between cross types within the BC class in the frequency of reduced testes (χ^2^= 7.0165, p=0.0714). Similar to the abnormal tergite results, BC males suffered the greatest frequency of abnormal testes; nearly 1/3 of all BC males exhibited reduced testes (31.8%) whereas few were observed in F1 males (3.1%, p<0.0001) and none in PS males (0%, p<0.0001). The frequency of abnormal testes in F1 and PS males was also significantly different (p<0.0001). In sum, our results indicate that developmental abnormalities represent an additional, cost to hybridization over multiple generations.

### Fitness Component Assay 5 - Flight Performance

The majority of flies responded to the stimuli and moved into the wind tunnel, against the headwind, at least one chamber. However, BC flies were less likely than F1 or PS flies to enter the first chamber (PS= 84.1% and 74.67%, F1= 80.99% and 75.36% but only 68% and 56% for BC males and females respectively), indicating a reduced response to stimuli in BC flies. 

**Figure 5:**
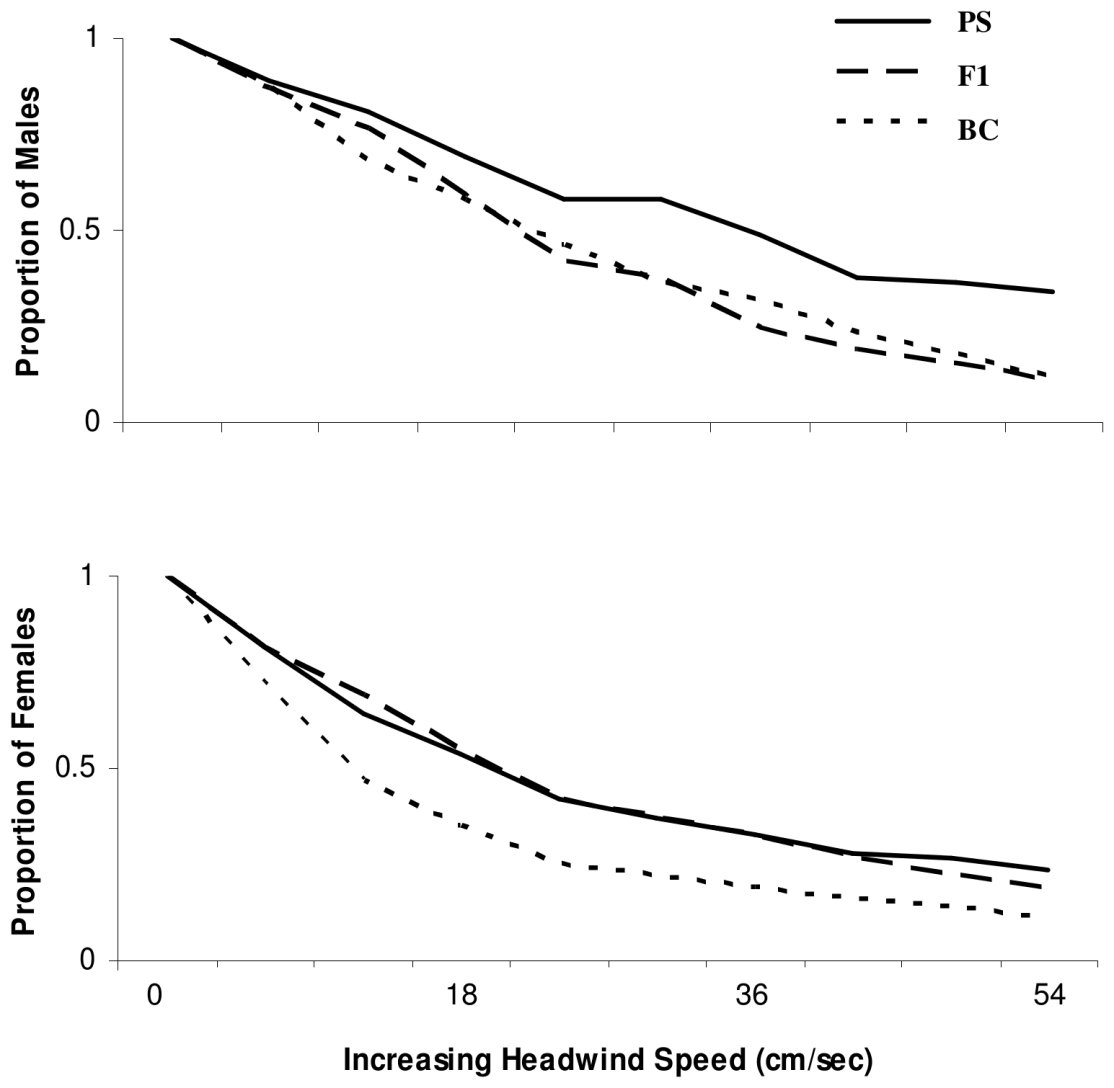
Upwind flight performance. Parental species (PS; *Drosophila pseudoobscura* and *D. persimilis*), F1 hybrid (F1), and backcross (BC) male (above) and female flies (below). Line shows the proportion of individuals that were able to fly into an increasing headwind.

The flight performance distribution for the PS males differed significantly from both the F1 and BC males (p<0.0167 and p<0.025, respectively). This difference was most pronounced at the strongest headwind speeds, where PS males had a higher frequency of individuals overcoming the strongest headwind to reach the final chambers (Figure 5). F1 and BC males did not differ in flight performance distribution (p>0.05). 

In contrast, the PS and F1 females did not differ in their flight performance (p>0.05). However, both PS and F1 females were significantly better at upwind flight than BC females (p<0.025 and p<0.0167, respectively). In this case, both PS and F1 females flew better at all wind speeds than BC females (Figure 5). In sum, this means that there are costs to hybridization that are manifest as both a reduction in response to attractants (BC flies) and in upwind flight ability (F1 and BC flies).

### Composite Estimate of Relative Fitness

We estimated the relative, composite fitness effects associated with hybridization for each sex for both the direct and indirect fitness traits, as well as the average fitness effect of hybridization for these two categories (Table 2). These composite estimates were then combined across sexes generating a within-generation estimate from the F1 and BC classes separately, and then combined these into a multi-generational index of the cost of hybridization (Table 2). Combining the results for our suite of fitness assays revealed that F1 hybrids were only ~31% as fit as parental species while the composite estimate for backcross offspring indicates a stronger cost to hybridization; backcross progeny are only ~11% as fit as the parental species. This general pattern is also observed even when examining the average fitness effects for both the direct and indirect fitness categories. When we combined estimates from these two generations, we found that the total, two-generational cost of hybridization was severe, a loss of ~ 96% (4% as fit) of fitness relative to purebred parental species. In both generations, hybrid origin males suffered a greater fitness decrement than females. F1 males have zero fitness driven by hybrid sterility while females lost 37% of their fitness relative to purebred parental species flies. A similar pattern was seen in BC flies as males were 3% as fit and females 19% as fit as their parental species counterparts.

**Table 2:** Relative fitness for each of the direct and indirect fitness components for males and females, as well as the average fitness effects and composite fitness effects for both fitness categories in the F1 and BC generations.

Fitness Component											
		Direct						Indirect			
Generation		Competition	Sterility*	Fecundity	Average	Composite		Dev. Abnormalities	Flight Performance	Average	Composite
F1											
males		0.668	0.000	N/A	0.334	0.000		0.985	0.331	0.658	0.326
females		0.668	0.835	1.372	0.958	0.765		1.000	0.829	0.915	0.829
BC											
males		0.650	0.209	N/A	0.430	0.136		0.721	0.349	0.535	0.252
females		0.650	0.517	1.220	0.796	0.410		0.952	0.474	0.713	0.451
		Composite Estimates									
Generation		Single-generation		Multi-generation							
F1		0.317									
males		0.000									
females		0.634		0.035	F1 * BC						
				0.000	males						
BC		0.110		0.117	females						
males		0.034									
females		0.185									

Lower table indicates the combined, composite fitness effect of hybridization for individual sexes, as well as the combined single and multi-generation composite estimates. Relative fitness was calculated as the mean of the hybrid divided by the mean of the parent. Competition values refer to the relative fitness in number of offspring produced in the High competition assays against the competitor ORPX. * Sterility data calculated from [41].

## Discussion

The cost of hybridization is rarely estimated using multiple fitness components or from multiple generations of hybrid and backcross progeny [7,25,32,33,36]. Although challenging, making such measurements is important as some costs of hybridization may be subtle, environmentally dependent, or manifest only in backcross progeny - yet the evolutionary fates of sympatric species are determined in large part by the relative fitness of offspring from intra- and interspecific matings [26]. Reduced hybrid fitness is predicted to select for the strengthening of species boundaries through the process of reinforcement, intensifying prezygotic isolation [1–3]. Here we have demonstrated varied, strong ecological and intrinsic sources of selection against hybridization in a well-studied species pair, *Drosophila pseudoobscura* and *D. persimilis*. 

We chose to assay representative fitness components that contribute to reproductive success via a diversity of direct and indirect processes throughout the lifecycle (Figure 2). Combining estimates across components from each sex, we found the relative fitness of F1 flies to be ~ 30% that of the parental species (Table 2); considerably less than the 50% fitness reduction caused by F1 hybrid male sterility typically thought to drive reinforcement in this system [9,34]. Interestingly, the average fitness decrement in backcross flies was even more severe, falling to < 15% the fitness of the parental species. Selection therefore goes beyond hybrid male sterility, affecting both male and female F1 hybrids, and backcross progeny. Thus, by our measures, hybrids and their backcross offspring are only ~5% as fit as *D. pseudoobscura* and *D. persimilis* - selection against hybrid and backcross progeny is so severe that it results in near complete reproductive isolation between species, although it takes two generations for this cost to be manifest fully. 

Caution should be applied, however, to not over-interpret the precise value of our composite fitness metric for several reasons. First, following Wiley et al. [7] and Moriarty Lemmon and Lemmon [25], all components and generations contribute equally to our composite fitness measure. For example, although fecundity and flight performance may contribute differently to actual fitness in nature, they are given equal weight in our composite fitness estimate. Second, some of our fitness components may be biologically or statistically correlated or dependent. Male sterility, for example, may derive from multiple sources including testis abnormality [41,82], yet sterility and frequency of developmental abnormalities are necessarily considered separately in our approach. Together, this means that our composite fitness estimate could unduly penalize hybrids by overweighing the contribution of a particular component or by assaying some non-independent costs more than once. 

Conversely, our composite estimates may not include important contributions from unmeasured fitness components. This is particularly true of traits related to sexual selection, which can play an important role in the reinforcement process [26,46,91], because it avoids the inefficiencies of post-zygotic natural selection [18,92,93]. Sexual selection accounts for the majority of the known fitness costs of hybridization for males in some species (e.g., 75% of reduction in hybrid male flycatchers [36], four times stronger sexual selection than natural selection against hybrid male chorus frogs [25]). We focused solely on natural selection against hybridization, in part because F1 males are sterile in this system and thus do not contribute to subsequent generations. They may, however, impose opportunity fitness costs by courting or mating with females. Additionally, male backcross progeny are fertile, creating the opportunity for sexual selection against hybridization through male courtship song parameters [94], cuticular hydrocarbon profiles [95] or other traits. The degree to which this occurs remains an open question.

To guard against being misled by such possible inaccuracies in our quantitative estimates of fitness costs, we compared informally variation among groups between direct and indirect fitness components and used the averages within these categories to calculate a second, more conservative, composite fitness measure. Both the direct and indirect fitness categories exhibited the same trends in direction and magnitude of costs among groups; males had lower fitness relative to females and BC flies were ~10-20% less fit than F1 flies; Table 2). Using the average of each category to calculate a second composite fitness measure yielded an estimate of relative hybrid fitness < 25% that of the parental species over two generations (combined for sex and generation, direct = 0.629, indirect= 0.705, composite of averages= 0.218). Thus, we are confident in our qualitative conclusion that there is strong, multifacted and multigenerational costs to hybridization in this system.

Our findings are generally consistent with Haldane’s rule of lower fitness in the heterogametic sex [49]. Although we did not find differences in sex ratio between parental species and F1 or BC flies in egg to adult viability (Assay 1), males suffered a disproportionate fitness cost. In addition to the well documented F1 male sterility [34,41], BC males had lower total fitness and substantially higher frequencies of developmental abnormalities than BC females (Table 2). Interestingly, we found no significant differences between the BC cross types in the frequency of abnormal tergites or testes, which contrasts with the expectation of X-chromosome related sterility effects [41]. Hybrid females, however, are not immune from costs associated with their hybrid origin. Although F1 females had slightly higher lifetime fecundity than PS females, this represents only a fleeting fitness advantage of hybridization as F1 females have poorer flight performance than PS adults, and the BC flies produced by F1 females are competitively inferior over the suite of fitness components we examined. F1 females were only 60% as fit as PS females over the suite of traits measured, a substantial cost not accounted for when considering only hybrid male sterility. Hence, there are substantial, cross-generational fitness costs for females resulting from hybridization as well. 

Studies of reinforcement and prezygotic isolation typically focus on single fitness components in F1 hybrids (e.g., [9–11]); ours is one of the few [7,25,33,37], to estimate several fitness components across the lifecycle for more than one generation of hybridization. We found F1 fitness to be nearly 35% lower than estimates derived from male sterility alone [9,34]. Interestingly, the fitness decrement in the first generation of backcross flies is even more severe, with the average fitness falling to <15% the fitness of parental species flies. The natural selection against hybridization we document, combined with the behavioral isolation associated with strong conspecific female choice [9], supports the conclusion that reinforcement is acting strongly in this system and may help explain the very low observed hybrid frequencies (0.0001% ) in nature [55,66]. But the more general conclusion from our work is that considering the full, multi-faceted and multi-generational costs of hybridization may allow for a greater role of reinforcement in the maintenance of species boundaries and diversification in an array of systems, especially those lacking extreme fitness reducing traits such as male hybrid sterility.

## Supporting Information

File S1 Supporting tables.
(DOC)
