# Supplementary material for: Multifaceted, Cross-Generational Costs of Hybridization in Sibling Drosophila species
Source: PLoS One. 2013 Nov 12;8(11):e80331. doi: 10.1371/journal.pone.0080331 (PMC3827178; doi:10.1371/journal.pone.0080331)
Supplement: File S1 — Supporting tables. (DOC) [file pone.0080331.s001.doc]

**Supplemental Document.**

**Table S1:** Lineages and populations used in the experiments; all but one (*) were used to generate the synthetic outcross populations for each parental species (PS) *Drosophila persimilis* and *D. pseudoobscura* stock populations. Lineage numbers refer to reference number at Drosophila Species Stock Center. MSH provided by M. Noor. Genetic status from Stock Center descriptions ^1^Isofemale, ^2^Inbred

***D. persimilis* Locality Mutation**

111.01 Quesnal, Canada

111.24 McDonald Ranch, CA

111.17 Port Townsend, WA

111.35 Mount San Jacinto, CA

111.41 British Columbia, Canada

111.46^2^ Mount St. Helena, CA

111.48^2^ Mount St. Helena, CA

MSH Mt. St. Helena, CA

111.50^1^ Santa Cruz Island, CA

111.51^1^ Santa Cruz Island, CA

111.55 Santa Cruz Island, CA Sepia

***D. pseudoobscura* Locality Mutation**

121.09 unknown Vermillion

121.94 Mesa Verde, CO

121.105^1^ Mesa Verde, CO

121.109^1^ Bonito Lake, NM

121.111^1^ Provo, UT

121.114^1^ Provo, UT

121.117^1^ Tucson, AZ

121.118^1^ Chiracahuas Mountains, AZ

121.150^1^ Organ Pipe National Monument, AZ

121.151^1^ Flagstaff, AZ

121.153^1^ Wilcox, AZ

*121.21 unknown Orange, Plexus

*This is OR-PX, the mutant used in the larval competition experiment; it was not used to create the PS stocks.

**Table S2:** Means and standard deviation (SD) for each species/cross directions for PS, F1, and BC. *Drosophila pseudoobscura* abbreviated at PSE. *D. persimilis* abbreviated as PER. Statistical tests examining the data and results comparing cross directions/species with population class (PS, F1, BC). Significant results in italics.

All crosses given as Male x Female.

For BC crosses, F1 female cross direction is abbreviated as follows:

PSE x PER = A

PER x PSE = B

**Egg to Adult Viability**

**PS/F1**

Class N Mean SD

PSE 3 42 2.082

PER 3 44.667 4.359

PSE x PER 9 42.333 3.122

PER x PSE 9 40.444 6.425

**Statistics**

**ANOVA w/in class**

DF F ratio p

PS 1,4 0.9143 0.3931

F1 1,16 0.6293 0.4392

**PS/BC**

Class N Mean SD

PSE 9 38.000 9.823

PER 9 37.889 6.153

PSE x A 9 33.111 7.524

PSE x B 8 32.625 5.290

PER x A 9 31.667 8.470

PER x B 9 27.000 12.00

**Statistics**

**ANOVA w/in Class**

DF F ratio p

PS 1,16 0.0008 0.9774

BC 3,31 0.9100 0.4475

**Data characterization for across Class analysis**

Levene’s test

F ratio DF p

F1 1.7079 1, 22 .2047

BC 0.3037 1, 51 0.5840

Shapiro-Wilk Test on residuals

W p

F1 0.9599 0.4368

BC 0.9177 *0.0014*

**Larval Competition Against ORPX**

Class N Mean SD proportion SD

**High**

PER 18 70.222 11.389 0.702 0.114

PER x A 9 48.778 12.891 0.488 0.130

PER x B 9 43.333 6.708 0.433 0.067

PER x PSE 9 61.000 21.702 0.610 0.217

PSE 18 69.111 9.934 0.691 0.099

PSE x A 9 50.889 15.292 0.509 0.153

PSE x B 9 44.889 10.130 0.449 0.101

PSE x PER 8 26.625 20.805 0.266 0.209

**Med**

PER 18 34.167 6.776 0.683 0.135

PER x A 9 29.444 7.634 0.589 0.152

PER x B 9 21.333 5.025 0.427 0.100

PER x PSE 9 28.222 5.118 0.564 0.102

PSE 18 37.333 5.099 0.747 0.102

PSE x A 9 26.667 9.859 0.533 0.197

PSE x B 9 22.000 6.633 0.440 0.133

PSE x PER 9 21.778 11.245 0.436 0.225

**Low**

PER 18 19.333 4.602 0.773 0.184

PER x A 9 16.222 3.032 0.649 0.121

PER x B 9 12.111 3.140 0.484 0.126

PER x PSE 9 15.556 6.126 0.622 0.245

PSE 18 19.778 4.037 0.791 0.161

PSE x A 9 16.778 3.492 0.671 0.140

PSE x B 9 16.222 2.587 0.649 0.103

PSE x PER 9 9.556 6.366 0.382 0.255

**Statistics**

**ANOVA w/in Class**

DF F ratio p

PS High 1,34 0.0973 0.757

F1 High 1,15 11.043 *0.0046*

BC High 3,32 0.7928 0.5069

PS Med 1,34 2.5100 0.1224

F1 Med 1,16 2.4488 0.1372

BC Med 3,32 2.3996 0.0861

PS Low 1,34 0.0949 0.7600

F1 Low 1,16 4.1509 0.0585

BC Low 3,32 4.4431 *0.0102*

**Data characterization for across Class analysis**

Shapiro-Wilk Test on residuals

W p

0.9941 0.3855

**Fecundity**

**Total Counts Longevity (wks) Per capita**

Class N Mean SD Mean SD Mean SD

PSE 3 3435.67 1757.91 8.933 2.386 35.190 13.306

PER 3 1355.00 329.00 8.233 1.570 15.035 0.288

PER x PSE 3 2309.67 533.821 7.267 2.084 38.526 7.672

PSE x PER 3 4262.67 708.249 10.167 0.611 43.902 6.977

PER x A 3 2184.33 542.21 9.800 0.854 22.451 2.988

PER x B 3 1885.67 1047.90 9.200 0.794 19.506 9.943

PSE x A 3 3400.67 869.51 9.833 0.306 32.928 11.107

PSE x B 3 4217.33 273.75 10.900 0.985 39.363 5.352

**Statistics**

**ANOVA w/in Class**

**Counts Longevity Per capita**

DF F ratio p F ratio p F ratio p

PS 1,4 4.0605 0.1141 0.1802 0.6930 6.8802 0.0586

F1 1,4 14.5473 *0.0189* 5.3491 0.0818 0.8060 0.4201

BC 3,8 6.3429 *0.0165* 2.4759 0.1358 3.9276 0.0541

**Data characterization for across Class analysis**

Levene’s test

F ratio DF p

Total Eggs 0.5589 2, 21 0.5801

Per Capita 1.4418 2, 21 0.2590

Longevity 3.1861 2, 21 0.0619

Shapiro-Wilk Test on residuals

W p

Total Eggs 0.945 0.2107

Per capita 0.9631 0.5055

Longevity 0.975 0.7864

**Frequency Developmental Abnormalities**

**Tergites Testes**

Class N Mean SD Mean SD

PSE 9 0.00000 0.00000 0.00000 0.0000

PER 9 0.00327 0.00980 0.00000 0.0000

PER x PSE 10 0.00000 0.00000 0.00000 0.0000

PSE x PER 8 0.00000 0.00000 0.06921 0.0788

PER x B 9 0.11963 0.10778 0.33262 0.2389

PER x A 8 0.05648 0.08025 0.38467 0.2182

PSE x B 9 0.02634 0.04701 0.27668 0.2135

PSE x A 8 0.03868 0.07164 0.28142 0.1950

**Statistics (Logistic Regression Analysis)**

**Tergites Testes**

DF χ^2^ p DF χ^2^ p

PS 1 1.3008 0.2541 1 - -

F1 1 - - 1 19.2986 <*0.0001*

BC 3 12.7026 0.0053 3 7.0165 0.0714
